# Reference letters for subspecialty medicine residency positions: are they valuable for decision-making? Results from a Canadian study

**DOI:** 10.1186/s12909-020-02270-7

**Published:** 2020-10-07

**Authors:** Deepti Chopra, Mala Joneja, Gurjit Sandhu, Christopher A. Smith, Catherine M. Spagnuolo, Lawrence Hookey

**Affiliations:** 1 Department of Medicine, Queen’s University, Kingston, ON Canada; 2 Departments of Surgery & Learning Health Sciences, University of Michigan, Ann Arbor, MI USA; 3 Division of Gastroenterology, Hotel Dieu Hospital, 166 Brock Street, Kingston, Ontario K7L 5G2 Canada

**Keywords:** Reference letter, Letter of recommendation, Residents, Medicine subspecialty match

## Abstract

**Background:**

The letter of recommendation is currently an integral part of applicant selection for residency programs. Internal medicine residents will spend much time and expense completing sub-specialty away electives to obtain a letter of recommendation. The purpose of this study was 1) to examine a large sample of reference letters in order to define essential components of a high-quality letter, and 2) to elucidate the relationship between quality of reference letter and the letter writer.

**Methods:**

We conducted a two-phase study. In phase one, a large sample of letters of recommendation was examined using an audit tool as a coding framework. A 5-point composite endpoint of high-quality letter components was subsequently developed. In phase two, program director letters were compared to non-program director home institution and non-home institution elective letters based on inclusion of components of the 5-point composite endpoint using Chi square testing.

**Results:**

715 letters were examined (398 non-program director home institution letters, 201 program director letters, and 116 non-home institution elective letters). High-quality letter components were: nature of relationship, duration of relationship, In Training Evaluation Report information, research involvement and comments on areas for improvement. Program director letters had a significantly higher proportion (10.4%) of all 5 high-quality components, compared to 0% in both non-program director home institution letters and elective letters (*p* < 0.001). A significantly higher proportion of program director letters had 4–5 high-quality components (62.5%) compared to 2% of non-program director home institution letters and 0% of elective letters (*p* < 0.0001).

**Conclusions:**

Letters of recommendation from elective rotations are of the poorest quality and such rotations should not be pursued for the sole purpose of obtaining a letter. The low quality of elective letters leads to the recommendation that writers should decline to write them, programs should not require them and trainees should not request them. Program directors write the highest quality letters and should be a resource for faculty development. Clinical supervisors can use the 5-point composite endpoint as a guide when writing letters for applicants.

## Background

The Letter of Recommendation (LOR) is an integral part of applicant selection for internal medicine subspecialty training programs. LORs are considered one of the most important factors in ranking candidates to postgraduate subspecialty programs and may be helpful in predicting residency retention rates [1–5]. However, the writing of LORs has also been described as a process that very few understand [6] and the role of the LOR has been deemed worthy of further examination.

LORs have been found to lack a meaningful comparison of the applicant to peers and often fail to include concrete examples demonstrating applicant performance [7]. LORs will often include “lengthy reiterations of already available objective data” and render all applicants “excellent,” making it extremely difficult to discriminate between them [8, 9]. In response to these noted shortcomings, program directors and medical educators are often asked to comment on what could make LORs more helpful. Researchers have examined, in a limited fashion, the role of the letter writer [10], key elements and phrases in letters and correlations between LORs with objective data [11, 12].

Our previous research which included a survey of Canadian internal medicine program directors has provided some insight into the challenges with letter writing and interpretation. Program directors (PDs) felt LORs lacked a common vocabulary and format and that referees often used variable rating scales or similar statements that did not differentiate between candidates. One example of this is the observation that a significant proportion of residents appeared to be in the top 5% of applicants [13]. The Canadian internal medicine PDs also suggested that there may be certain reference letter features that are more useful than others, and this led us to explore LORs further [13].

We partnered with the Canadian Resident Matching Service (CaRMS) to evaluate LORs for internal medicine residents applying to subspecialty training programs. The study objectives were to: 1a) elicit the key components of letters which affect quality through a large audit of LORs to subspecialty internal medicine programs in Canada between 2011 and 2014 and b) subsequently define the components of a high-quality letter; and 2) compare PD letters to non-PD home institution and non-home institution elective letters (elective letters) based on the inclusion of high-quality components.

## Methods

Ethics approval was obtained from the Queen’s University Health Sciences & Affiliated Teaching Hospitals Research Ethics Board and the Canadian Resident Matching Service (CaRMS) Board.

This study was designed in two phases.

### Phase 1: creation of composite endpoint for a high-quality LOR

The objective of phase one was to identify common components present in LORs and then by consensus, select the components that are necessary for a LOR to be deemed high-quality.

### Common components of LORs

CaRMS provided 738 de-identified reference letters that had been submitted to subspecialty internal medicine programs (for example, gastroenterology, rheumatology, cardiology etc.) over a 3-year period (2011–2014). Letters had been randomly selected by staff at CARMS and the distribution across specialties correlated well with the overall distribution for the match from 2011 to 2014. An audit tool was established based on a literature review of LORs in an effort to collect quantitative and qualitative data, including information and examples according to categories of CanMeds roles and details of letter writer (type of supervisor, relationship to trainee, duration of relationship).

The research assistant conducted content analysis [14, 15] using open coding of the reference letters with the audit tool as a coding framework to identify common components. She independently reviewed 10% of reference letters. The research team subsequently reviewed the same data set. Discrepancies among the team were resolved and the audit tool was refined for clarity. The resultant iteration of the audit tool provided a clear list of components for ongoing coding (Appendix). The research assistant was not associated with any resident or faculty investigators to address reflexivity and mitigate bias [16].

### Defining a high-quality letter: construction of the composite endpoint

**Fig. 1:**
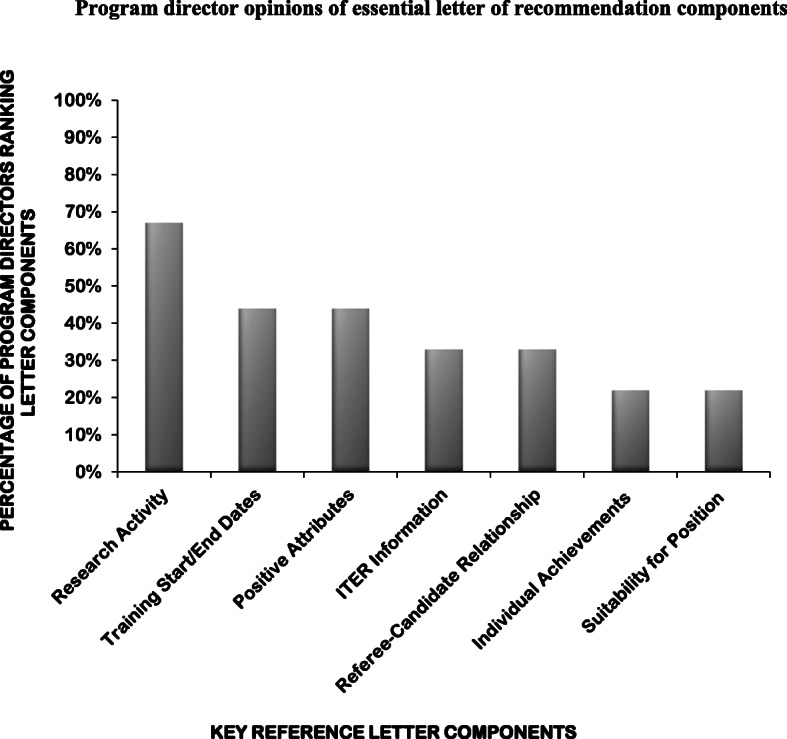
Program director opinions of essential letter of recommendation components

A composite endpoint consisting of five items was created to define a high-quality LOR. The composite endpoint was created by selection of the five most valuable components of LORs from the list of identified common components, based on: 1) the opinions of Canadian internal medicine program directors obtained through a survey [13] (Fig. 1), 2) results of a detailed literature review, and 3) consensus from a panel of local medical educators, including subspecialty medicine program directors and the dean of postgraduate education.

### Phase 2: reference letter comparison based on referee type

Letters from PDs (PD letters) were compared to (i) letters by non-PDs from the home school (non-PD home institution letters), and (ii) letters obtained on elective rotations (elective letters). Letters were compared with respect to the inclusion of individual components of the 5-point composite endpoint. The inclusion of In Training Evaluation Report (ITER) information was identified as one of the components of the composite endpoint. Upon reflection that elective letters were unlikely to include this data, a separate comparison was also performed, removing the ITER component and leaving a 4-point composite endpoint. Analysis was also performed to assess if a writer had met the criteria for a good to high-quality letter by including a majority of the components.

Chi square testing was performed to compare frequencies using SPSS Statistics, version 24 (IBM, Armonk, New York). A sample size of 105 per group was necessary to demonstrate a 20% difference in proportion of high-quality letters using three-step pairwise comparison in an ANOVA.

## Results

### Phase 1: creation of composite endpoint for a high-quality LOR

The components necessary for a high-quality letter were determined to be:

1. nature of relationship between the referee and candidate, such as rotation supervisor, research supervisor, and/or program director
2. duration of this relationship-measured in weeks.
3. inclusion of In Training Evaluation Report (ITER) information
4. research involvement comments, often outlining role in specific projects
5. area for improvement or critique comments.

Given the possibility that only PDs would have ready access to ITER information, we also performed the analysis excluding this component, using a 4-point composite endpoint.

### Phase 2: reference letter comparison based on referee type

738 letters were reviewed, with 715 included in the final analysis. Twenty-three were excluded due to duplicates or redaction beyond ability to extract meaningful information. The distribution of reference letters was: 398 non-PD home institution letters (55.7%), 201 PD letters (28.1%), and 116 elective letters (16.2%). Letters were written for 206 applicants, with a range of 1–5 letters per applicant reviewed. Applicants were applying for 9 specific sub-specialties, with good representation of the breath of sub-specialty internal medicine. There were 113 letters for which the specialty being applied for was not evident. There were no letters of application for allergy, infectious disease, or endocrinology. Letter writers were affiliated with 33 different institutions within Canada and the United States.

With respect to the primary outcome (inclusion of all 5 high-quality components of the 5-point composite endpoint), PD letters had a significantly higher proportion of letters that met this target (10.4%), compared to 0% in both non-PD home institution letters and elective letters (*p* < 0.001) (Table 1). Using the “non ITER” 4-point composite endpoint, PDs again had a significantly higher proportion with all four components (12.4%), compared to non-PD home institution letters (1.3%) and elective letters (0%) (Table 2).

**Table 1:** Comparison of composite score based on the 5-point composite endpoint (ITER included) across letter writers

		Letter writer			*P* value
		Non-program director home institution letters, *n* = 398	Program director letters, *n* = 201	elective letters, *n* = 116	
Composite score	0	9	0	6	< 0.0001
	1	67	5	50	
	2	172	17	47	
	3	142	54	13	
	4	8	104	0	
	5	0	21	0	

Findings are significant for subgroup analysis of home versus elective as well (*p <* 0.0001)

**Table 2:** Comparison of composite score based on the 4-point composite endpoint (ITER excluded) across letter writers

		Letter writer			*P* value
		Non-program director home institution letters, *n =* 398	Program director letters, *n =* 201	elective letters, *n =* 116	
Composite score	0	9	0	6	< 0.0001
	1	68	5	51	
	2	174	49	47	
	3	142	122	12	
	4	5	25	0	

Findings are significant for subgroup analysis of home versus elective as well (*p <* 0.0001)

When evaluated for having a majority of the components of the 5-point composite endpoint, a significantly higher proportion of PD letters had 4–5 components (62.5%) compared to 2% of non-PD home institution letters, and 0% of elective letters (*p* < 0.0001) (Table 1). Of PD letters, 94% contained 3 or more components, compared to 37.7% of non-PD home institution letters, and 11.2% of elective letters (*p* < 0.0001) (Table 1). Likewise, for the 4-point composite endpoint, 73.1% of PD letters had 3–4 components compared to 36.9% of non-PD home institution letters and 10.3% of elective letters (*p <* 0.0001) (Table 2). Further breakdown of letter components is seen in Table 3.

**Table 3:** Prevalence of each component of the 5-point composite endpoint across letter writers

		Letter writer			*P* value
		Non-program director home institution (*n =* 398)	Program director (*n =* 201)	Elective supervisor (*n =* 116)	
Any mention of	Nature of relationship	379	194	104	0.025
	Duration of relationship	323	181	82	< 0.0001
	ITER comments	7	153	2	< 0.0001
	Research involvement	220	139	61	0.002
	Area of improvement/ critique	23	36	3	< 0.0001

## Discussion

Letters of recommendation continue to be a key component of the modern fellowship application [6]. However, this current study of over 700 LORs reveals a significant variability in letter quality. Variability in letter quality appeared dependent on the relationship between the writer and applicant. Not surprisingly, PDs’ letter writing practice is consistent with their recommendations. This is demonstrated by the fact that PD letters had the highest proportion of high-quality letters (10.4% included all 5 components of the 5-point composite endpoint and 94% had 3 or more components), while letters from elective supervisors were of much lower quality, with 0% having all 5 components of the 5-point composite endpoint, and only 11.2% having 3 or more components.

Although there is existing literature examining LORs, the current study adds to this in several respects. Most studies to date have looked at trainees in the United States medical training system [10, 11, 17], while the current study examined letters from across Canada, with a different healthcare and education system, thus adding the experience of another country and enriching the data pool. This study also highlights the relationship between letter quality and *letter writer*, leading to opportunity for possible faculty development with a goal of improving letter quality. Lastly, it brings forth the issue of elective rotations, and their role as auditions for position, and whether letters of recommendation should be a part of key outcomes of this effort, particularly in light of the poor quality of the letters that come from the experience.

### The letter writer

Program directors practice what they preach. They understand the value of specific components of LORs and include these components with the highest frequency. This indicates that PDs are an untapped resource for providing faculty development to all clinical supervisors who are potential letter writers. In stark contrast to PD letters, LORs from elective rotation supervisors were of the lowest quality. One possible reason for this finding is that a short amount of time spent with an unknown trainee does not provide sufficient data to allow the supervisor to be comfortable in rating the trainee in key domains. As many residents will seek out elective rotations at other sites with the intention of not only introducing themselves, but also to obtain a LOR from this site, the overall poor quality of these letters calls into question at least one element of the perceived value of these rotations.

### Elective rotations

While letters from elective supervisors represent approximately 15% of overall letters in our sample, their utility appears very limited; a meagre 11.2% met the criteria for a good to high-quality letter by including 3 or more components of the 5-point composite endpoint. Questions raised by this include 1) why would trainees get these letters, and 2) did this study miss some important aspect of the letter that would make letters from electives important? While our data suggest elective letters are not of good quality, and thus seemingly not useful overall, they may be a necessity for a particular institution, regardless of how non-specific the information within them, as suggested by recent papers from the surgery field [18, 19]. These could be considered letters from an ‘audition elective’- a term used to describe a clinical elective taken by trainees to distinguish themselves from their peers in order to improve their chances of being selected by a residency program [20]. Internal medicine residents will spend a great amount of time and expense completing sub-specialty audition electives to improve their chances of success in the subspecialty match, possibly via obtaining a strong reference letter from their elective supervisors. As we have shown the LORs from elective rotations to be of low quality, this calls into question the value of these rotations, given their associated costs. With the current travel restrictions related to the COVID-19 pandemic, completion of these away rotations may be even more difficult. This is consistent with other studies questioning the value of the ‘away rotation’ and that the value of such rotations should not depend solely on the obtaining of a LOR [21].

The current study does have certain limitations. While exposing key differences in letter quality according to the letter writer, further exploration of this data is somewhat limited. To ensure anonymity, the letters underwent redaction prior to release by CaRMS, with demographic data including age, gender, and candidate home institution name being removed. Moreover, we were unable to correlate these findings with subspecialty match or residency performance results, thus limiting us to examining the process rather than outcomes that may be of interest to a PD. We put a lot of weight on the opinions of internal medicine PDs, largely because of their experience in both writing and reading letters of recommendation. However, there are other members of selection committees, whose views and opinions of a quality letter may differ. In consideration of this, our composite endpoint was developed with input from the internal medicine PD survey, but also with input from a panel of academic internal medicine subspecialists who sit on various residency training selection committees. Lastly, the current study only looked at letters for application to the subspecialty internal medicine match and results may not be transferable to programs accepting medical students into their first year of residency training (PGY-1).

## Recommendations

1. LORs from elective rotations: We recommend that letters from elective rotations should not be mandatory for consideration of acceptance into a program, as well as consideration of a faculty wide approach/position that writers will not provide letters based solely on elective rotations. This recommendation would presumably lead to trainees asking for letters from other sources, likely faculty that know them better and thus can provide a higher quality letter.
2. Faculty development: Program directors are a potential resource for faculty development and can provide guidance to letter writers based on their experience and expertise in postgraduate medical education. Institutions should approach program directors for faculty development strategies that will lead to improved quality of LORs.
3. Letter writers: Clinical supervisors who are writing letters for applicants in the Medicine Subspecialty Match can use the components of the 5-point composite endpoint as guide for the inclusion of important components (relationship between the referee and candidate, duration of relationship, inclusion of In Training Evaluation Report (ITER) information, research involvement and comments on areas for improvement). Also, if a supervisor does not feel they can comment on one or more of these components, then it would be acceptable to decline a request for a LOR from an applicant.
4. Letter readers: Similarly, those reviewing applicants’ portfolios can use the components of the 5-point composite endpoint to judge the quality of the letter.
5. Applicants: Applicants should be aware of what program directors value in LORs and should direct their requests to supervisors who are appropriate and capable of commenting on the key components.

## Conclusions

In conclusion, LORs continue to be variable in quality. There continues to be a quality gap in LORs for subspecialty medicine positions, in particular with respect to letters from elective rotations. Program directors write the highest quality LORs and should be a resource for faculty development. LORs from away rotations are of the poorest quality and such rotations should not be pursued for the sole purpose of obtaining a letter. Future studies are needed to assess outcome and to study clinical performance of trainees who have letters from elective rotations. Future research could involve surveying trainees and program directors for deeper insight into what drives LORs and opportunities for improving them.

## Data Availability

The datasets used and/or analysed during the current study are available from the corresponding author on reasonable request.

## Appendix

### Letter of Reference Audit Tool – Data Collection Form

Y = Yes.

N = No.

NC = No comment.

EXCEL FILE HEADINGS:

1. Applicant letter code
2. Name of applicant’s institution
3. Letter from applicant’s home institution Y/N
4. Descriptor(s) used to describe resident (general opening descriptor) Y/N
  - What descriptors were used?________________________________
5. Used % to rate candidate? Y/N
  - State %____________
6. Qualitative statement used to compare to peers Y/N
  - If yes ____________________
7. Did the resident see his/her letter Y/N/NC
8. Nature of referee-candidate relationship
  - 1 = program director
  - 2 = elective supervisor
  - 3 = research supervisor
  - 4 = clinical supervisor
  - 5 = combination of above
9. Duration of relationship stated Y/N
  - If yes, what was the duration (days) ______
10. Nature of activity
  - 1 = inpatient
  - 2 = outpatient clinic
  - 3 = research
  - 4 = program director
  - 5 = combination of above
11. General statement included e.g. excellent resident Y/N
  - If yes ________________________________

Descriptors stated under the following categories: Y/N, if yes then state the specific comment included in the reference letter

12. Knowledge
13. Problem solving and patient management
14. Behaviour and attitudinal skills
15. Communication skills
16. Ability to work in a team
17. Leadership
18. Motivation and punctuality
19. Sense of responsibility
20. Procedural skills specific to the discipline
21. Were procedural skills observed Y/N
  - What information was the assessment based on e.g. file review
22. Patient advocacy Y/N
23. Examples cited from:
  - D = direct ITER comments
  - S = summary of ITER comments
  - N = no ITER comments
  - O = other source of comments
24. Special qualities commented on (future potential etc.) Y/N
  - If yes ________________________
25. Individual achievements/unique contributions (chief resident, volunteering etc.) Y/N
  - If yes ________________________
26. Research Y/N
  - If yes record the following:
    i. Stage of research e.g. submitted, in progress, accepted abstract or poster_______________________________
    ii. Number of publications _________
27. Awards Y/N
  - If yes ______________________
28. Extracurricular activities Y/N
  - If yes ______________________
29. Education involvement Y/N
  - If yes ______________________
30. Suitability for program comment Y/N
  - If yes ______________________
31. Area for improvements Y/N
  - If yes ______________________
32. Specific critiques Y/N
  - If yes ______________________
33. CaRMS format for reference letter used Y/N
34. Number of CaRMS guidelines followed _______
35. Other comments

## Abbreviations

LOR: Letter of recommendation
PD: Program director
CaRMS: Canadian Resident Matching Service
ITER: In Training Evaluation Report
MSM: Medicine Subspecialty Match
